# A novel *PTCH1 *germline mutation distinguishes basal cell carcinoma from basaloid follicular hamartoma: a case report

**DOI:** 10.1186/1752-1947-3-52

**Published:** 2009-02-09

**Authors:** Ali Hellani, Hiba Baghdadi, Nidal Dabbour, Nidal Almassri, Khaled K Abu-Amero

**Affiliations:** 1 PGD laboratory, Saad Specialist Hospital, Al-khobar, 31952 Saudi Arabia; 2 Dermatology Department, Saad Specialist Hospital, Al-khobar, 31952 Saudi Arabia; 3 Pathology and Laboratory Medicine Department, Saad Specialist Hospital, Al-khobar, 31952 Saudi Arabia; 4 Molecular Genetics Laboratory, College of Medicine, King Saud University, Riyadh, Saudi Arabia

## Abstract

**Introduction:**

Nevoid basal cell carcinoma syndrome is a rare autosomal dominant disorder characterized by numerous basal cell carcinomas, odontogenic keratocysts of the jaws and developmental defects. The disorder results from mutations in the *PTCH1 *gene.

**Case presentation:**

A 15-year-old boy presented to our dental clinic with multiple jaw cysts. The patient had broad confluent eyebrows, a broad base of the nose, frontal bossing and palmoplantar pits. Examination of the jaw cysts revealed many keratinizing cysts without granular cell layers a finding that raised the suspicion of nevoid basal cell carcinoma. Radiological examinations showed calcification of the falx cerebri, spina bifida, bifid thoracic ribs and frontal bossing. Histopathological examination showed basaloid proliferation in the upper dermis with follicular differentiation surrounded by a loose mucinous stroma and retraction artifacts. These features make it difficult to differentiate between nevoid basal cell carcinoma and basaloid follicular hamartoma, especially the presence of these findings on a non-hairy area. BCL-2 staining was positive in the periphery of the basaloid proliferation, which is typical of basaloid follicular hamartoma, and not in a diffuse pattern, which is typical of nevoid basal cell carcinoma. The proband's siblings and parents were healthy with no family history of this condition in the extended family. Since histology was equivocal and palmoplantar pits are seen in both basaloid follicular hamartoma and nevoid basal cell carcinoma, molecular genetic investigation was necessary to differentiate between the two potential diagnoses. After sequencing the entire *PTCH1 *gene, we detected a single nucleotide deletion (c.1291delC) in codon 431 of the PTCH protein, which resulted in a premature stop translation at residue 431. This *de novo *mutation was not detected in both parents and in 100 normal volunteers of matching ethnicity.

**Conclusion:**

Screening the *PTCH1 *gene for mutations helped to differentiate between basaloid follicular hamartoma and nevoid basal cell carcinoma and confirmed the diagnosis.

## Introduction

Nevoid basal cell carcinoma syndrome (NBCC; also known as Gorlin syndrome; MIM #109400) is a rare autosomal dominant disorder characterized by numerous basal cell carcinomas, odontogenic keratocysts of the jaws, and developmental defects such as bifid ribs, intracranial calcification, and polydactyly [1]. NBCC also predisposes individuals to a variety of low-frequency tumors such as ovarian fibroma, medulloblastoma, rhabdomyosarcomas, and cardiac fibromas [1]. Multiple organ systems may be impacted in NBCC. Abnormalities of the skin, the skeletal system, the genitourinary system, and the central nervous system (CNS) are the most common. The approximate prevalence is reported to be one case per 56,000–164,000 population. The prevalence is likely to be considerably higher in individuals younger than 20 years who present with basal cell carcinomas (BCC). The syndrome has been documented for 50 years, but recent developments in molecular genetics have dramatically increased our understanding of its pathophysiology. The disorder results from mutations in the *PTCH1 *gene, the human homolog of the *Drosophila *segment polarity gene, patched (MIM # 601309). *PTCH1 *has been mapped to 9q22.3-q31 and consists of 23 exons spanning approximately 50 kb and encoding a 1447-amino acid transmembrane glycoprotein. PTCH protein is involved in Sonic hedgehog (Shh) signaling, where it is thought to act as a receptor for Shh ligands [2]. An important clue to the understanding of PTCH function comes from the study of its interactions with another membrane protein smoothened (Smo). In the absence of Shh signal, PTCH represses the constitutive signaling activity of Smo, by forming a PTCH-Smo complex [2]. Pathogenic mutations in the *PTCH1 *gene result in the failure of PTCH to inhibit Smo, leading to the constitutive activity of the Shh signaling pathway [3]. The Shh signaling pathway has been implicated in the formation of embryonic structures and tumorigenesis [4]. Therefore, a disorder of this pathway could result in an abnormal body conformation and tumorigenesis as seen in NBCC patients. To date, over 100 *PTCH1 *germline mutations associated with NBCC have been reported, most (73%) identifying nonsense or frameshift mutations leading to the synthesis of a truncated PTCH protein [5]. These mutations appear to be mainly clustered into the large extra- and intracellular loops of the PTCH protein, but no apparent genotype-phenotype correlations have been established.

## Case presentation

**Figure 1:**
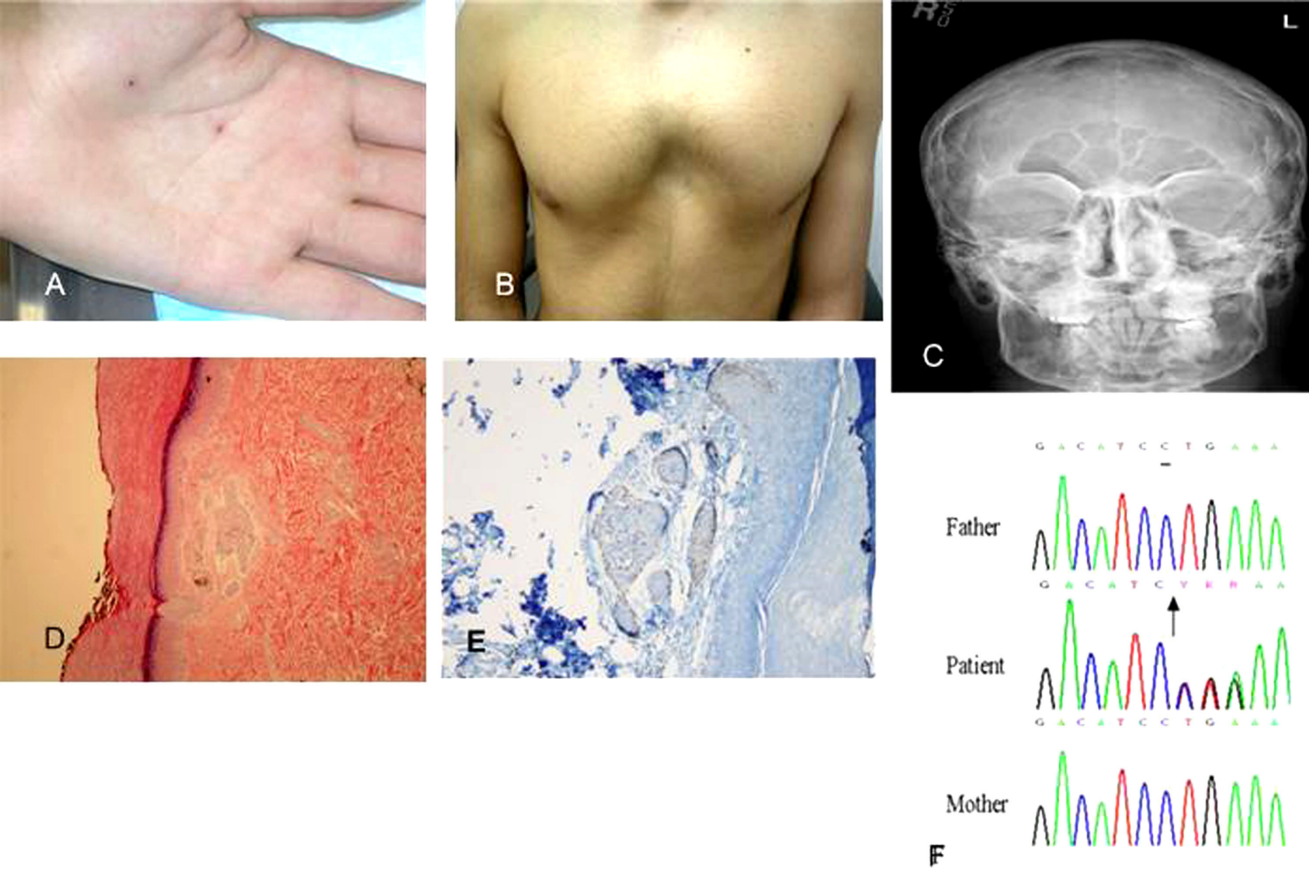
**(A) Palmoplantar pits biopsied and non-biopsied**. (B) Pectus excavatum. (C) Calcification of the falx cerebri. (D) The superficial dermis contains nests of basaloid tumor cells with peripheral palisading nuclei surrounded by mucinous stroma. (E) BCL-2 immunostaining illustrates focal brown staining of the tumor cells. Many of the tumor cells lack BCL-2 expression. (F) Chromatogram showing the forward sequencing results of the *PTCH1 *gene for the patient and his parents. The arrow indicates the position of the deleted nucleotide "C" in the patient. The sequence after the deleted "C" in the patient was deteriorated as expected when a sequence deletion is present.

A 15-year-old boy presented to our dental clinic because of multiple jaw cysts. The patient had the following clinical features: tall stature, broad confluent eyebrows, broad base of the nose, frontal bossing, palmoplantar pits (Figure 1A), and pectus excavatum (Figure 1B). Intellectually, the patient was normal with an above average intelligence level in school. Both parents and siblings were normal and there was no family history of such a condition or intellectual impairment in their extended family. Histopathological examination of the jaw cysts revealed keratinizing cysts lined by stratified squamous epithelium with variable cell thickness. Keratinization appeared to be abrupt with lack of a granular cell layer.

The presence of multiple jaw cysts devoid of a granular layer raised the suspicion of nevoid basal cell carcinoma. The patient had multiple palmoplantar pits. His radiological examinations revealed calcification of the falx cerebri (Figure 1C), spina bifida, bifid thoracic ribs and frontal bossing.

Histopathological examination showed nests of tumor cells in the superficial dermis. These nests were characterized by proliferation of basaloid cells with palisading nuclei at the periphery of the nests. The stroma around the tumor had loose mucinous changes and retraction artifacts (Figure 1D). This appearance can be seen in both NBCC and basaloid follicular hamartoma (BFH). The presence of retraction artifacts favors NBCC; however, the pattern of immunostaining for bcl-2 with only focal cellular staining rather than the diffuse pattern (Figure 1E) was more consistent with BFH [6].

Since the histology was ambiguous and palmoplantar pits are seen in both BFH and NBCC, molecular genetic investigation was necessary to differentiate between the two entities. In order to do this, genomic DNA was amplified and the entire coding region and the exon-intron boundaries of the *PTCH1 *gene were sequenced as detailed previously [7]. Briefly, *PTCH1 *sequences were amplified from 100 to 200 ng of DNA using specific primers (5 μM), dNTP (5 mM), PCR buffer 10×, and one unit of Expand Long *Taq *polymerase (Roche). Polymerase chain reaction (PCR) products were purified using a Qiagen purification kit and then assessed on the capillary electrophoresis bio-analyzer using the DNA 7500 chip. The purified PCR product was sequenced on an ABI 3130*xI *Genetic Analyzer using forward and reverse primers described previously [7]. After sequencing the entire *PTCH1 *gene, we detected a single nucleotide deletion (c.1291delC) in codon 431, which resulted in a premature stop translation at residue 431 (Figure 1F). Since the *PTCH1 *encodes 1447-amino acid transmembrane glycoprotein, the premature stop-codon found at codon 431 is expected to have a deleterious effect on protein structure and function. This *de novo *mutation (c.1291delC) was not detected in either parents or in 100 normal volunteers of matching ethnicity. This mutation is located in the extracellular domain of the PTCH protein, which is known to be an important domain that interacts with the Shh ligand [8] and is expected to inactivate the protein ability to bind the Shh ligand. As a consequence of mutation identification and confirmation of the NBCC diagnosis, the management protocol for this patient would be: (i) surgical excision of any basal cancer cells; (ii) avoidance of sun exposure and any kind of radiotherapy; (iii) annual follow-up for the development of skin lesions and (iv) genetic counseling.

## Discussion

Nevoid basal cell carcinoma syndrome is a rare autosomal dominant disease characterized by developmental abnormalities and tumorigenesis. Mutation of the *PTCH1 *gene is the molecular defect associated with this syndrome. According to the human genome mutation database [9], there are currently 224 mutations reported to date (database updated May 2008) in the *PTCH1 *gene. Here, we describe a patient who presented with a clinical picture that was consistent with NBCC, but overlapped with BFH. Therefore, molecular genetic study was necessary in order to differentiate between the two entities. *PTCH1 *was investigated because *PTCH1 *mutations are the typical cause of NBCC, but not of BFH syndrome, where the causative gene is not yet known. Sequencing the *PTCH1 *gene revealed the presence of a *de novo *mutation (c.1291delC) that was not detected in either parents or in 100 normal volunteers of matching ethnicity. According to the *PTCH *mutation database and human mutation database, the *de novo *mutation (c.1291delC) detected in our patient has not been reported previously. Since this mutation will introduce a premature stop-codon early in translation, we expect this mutation to have serious consequences on the formation of mature protein and ultimately it will have deleterious consequences on protein function.

NBCC is malignant so that early detection and confirmed diagnosis are of paramount importance and will help avoiding unnecessary radiotherapy treatment for brain tumors usually developed by these patients.

Molecular genetic testing will be helpful to confirm the diagnosis in patients with atypical phenotype or possibly for prenatal diagnosis. Molecular testing may be useful for infants of an affected patient who is too young to have developed diagnostic clinical findings. Additionally, analysis of the *PTCH1 *gene in NBCC will provide important information not only for genetic counseling, but also for further research of the correlation between *PTCH1 *genotype and NBCC phenotype. We recommend that any patient with multiple jaw cysts and or palmoplantar pits should be evaluated by a dermatologist to look for the clinical findings of Gorlin syndrome.

## Conclusion

Screening of the *PTCH1 *gene for mutations is of paramount importance in confirming the diagnosis for this autosomal dominant syndrome.

## Abbreviations

BFH: basaloid follicular hamartoma; CNS: central nervous system; NBCC: nevoid basal cell carcinoma syndrome; PTCH: patched homolog (Drosophila); Shh: Sonic hedgehog; Smo: smoothened

## Consent

Written informed consent was obtained from the parents of the patient for publication of this case report. A copy of the written consent is available for review by the Editor-in-Chief of this journal.

## Competing interests

The authors declare that they have no competing interests.

## Authors' contributions

AH was in charge of design and overall supervision of the study. HB carried out the sequencing of the gene while ND was involved in the clinical evaluation of the patient. NA was in charge of the histopathological evaluation of the patient while KKA performed the analysis of the genetic data and drafted the manuscript.
